# An Adaptable Human-Like Gait Pattern Generator Derived From a Lower Limb Exoskeleton

**DOI:** 10.3389/frobt.2019.00036

**Published:** 2019-05-14

**Authors:** Rafael Mendoza-Crespo, Diego Torricelli, Joel Carlos Huegel, Jose Luis Gordillo, Jose Luis Pons, Rogelio Soto

**Affiliations:** ^1^Tecnologico de Monterrey, Escuela de Ingenieria y Ciencias, Monterrey, Mexico; ^2^Neural Rehabilitation Group, Cajal Institute, Madrid, Spain; ^3^Center for Extreme Bionics, Massachusetts Institute of Technology, Cambridge, MA, United States

**Keywords:** gait, trajectory, ankle, step length, eigenvalue decomposition, key events, heel strike, toe off

## Abstract

Walking rehabilitation processes include many repetitions of the same physical movements in order to replicate, as close as possible, the normal gait trajectories, and kinematics of all leg joints. In these conventional therapies, the therapist′s ability to discover patient′s limitations—and gradually reduce them—is key to the success of the therapy. Lower-limb robotic exoskeletons have strong deficiencies in this respect as compared to an experienced therapist. Most of the currently available robotic solutions are not able to properly adapt their trajectories to the biomechanical limitations of the patient. With this in mind, much research and development is still required in order to improve artificial human-like walking patterns sufficiently for valuable clinical use. The work herein reported develops and presents a method to acquire and saliently analyze subject-specific gait data while the subject dons a passive lower-limb exoskeleton. Furthermore, the method can generate adjustable, yet subject-specific, kinematic gait trajectories useful in programming controllers for future robotic rehabilitation protocols. A human-user study with ten healthy subjects provides the experimental setup to validate the proposed method. The experimental protocol consists in capturing kinematic data while subjects walk, with the donned H2 lower-limb exoskeleton, across three experimental conditions: walking with three different pre-determined step lengths marked on a lane. The captured ankle trajectories in the sagittal plane were found by normalizing all trials of each test from one heel strike to the next heel strike independent of the specific gait features of each individual. Prior literature suggests analyzing gait in phases. A preliminary data analysis, however, suggests that there exist six key events of the gait cycle, events that can adequately characterize gait for the purposes required of robotic rehabilitation including gait analysis and reference trajectory generation. Defining the ankle as an end effector and the hip as the origin of the coordinate frame and basing the linear regression calculations only on the six key events, i.e., Heel Strike, Toe Off, Pre-Swing, Initial Swing, Mid-Swing, and Terminal Swing, it is possible to generate a new calculated ankle trajectory with an arbitrary step length. The Leave-One-Out Cross Validation algorithm was used to estimate the fitting error of the calculated trajectory vs. the characteristic captured trajectory per subject, showing a fidelity average value of 95.2, 96.1, and 97.2%, respectively, for each step-length trial including all subjects. This research presents method to capture ankle trajectories from subjects and generate human-like ankle trajectories that could be scaled and computed on-line, could be adjusted to different gait scenarios, and could be used not only to generate reference trajectories for gait controllers, but also as an accurate and salient benchmark to test the human likeness of gait trajectories employed by existing robotic exoskeletal devices.

## 1. Introduction

Gait rehabilitation usually implies specific routines consisting in numerous repetitions of the same exercises. One or several physical therapists (PTs) may be needed to help the patient re-create, as close as possible, the movements of a normal gait trajectory, e.g., two PTs supporting the right and left legs and a third PT stabilizing the trunk. This kind of therapy is time consuming, resource intensive, and can be frustrating for the patient. Assistance robots are well known to be precise, regular, and deterministic in supporting or substituting humans during repetitive tasks. Nevertheless, their safe and effective direct physical interaction with users is a critical factor, especially in rehabilitation scenarios. These scenarios still requires constant supervision from human operators.

One of the main challenges in rehabilitation robotics is to achieve a smooth and comfortable interaction between the user and the robotic device. Current exoskeletons are not flexible or compliant enough to adapt to biomechanical or cognitive limitations of each individual patient. As a consequence, patients are often constrained to follow rigid trajectories, replicating some normative kinematic reference. Some approaches do allow certain variations from these reference baselines, but no patient-specific adaptation mechanisms are currently available either in commercial or research devices. In order to compare and contrast previous developments in gait analysis, a review of current ankle trajectory models was conducted. The review includes, on the one hand, models employed to capture ankle motion, and on the other hand, models employed to execute the robotic ankle motion.

To emphasize the difference between mechanically generated trajectories and software generated ones, some mechanical systems are mentioned in the following lines. There are several groups that have tried to find a mechanical way to replicate human-like ankle joint trajectories while walking forward on flat ground. In Flores et al. (2013), Tsuge and Michael McCarthy (2016), Copilusi et al. (2014), Choi et al. (2017), Shao et al. (2016) they all synthesized trajectories starting from different number of bar linkage mechanisms for human gait rehabilitation and mobility enhancement. The biggest drawback to this kind of systems is the difficulty to make adjustments while it is working, thereby limiting the range of modifications that can be performed to generate different walking conditions, i.e., changing step length while the subject is running a test or adjusting the height of the exoskeleton segments to fit different subjects.

To perform assisted movement of the hip and knee joints, generate a path for the healthy ankle, and achieve that movement as close as possible to a specific reference trajectory (Banala et al., 2008), studied gait data. Tufekciler et al. found that there were specific variations in gait trajectories such as the peak values in position and velocity data, starting and ending of each trajectory, and some other extra parameters with fixed timing points (Tufekciler et al., 2011). Additionally, the reference trajectories proposed by Jezernik et al. (2004) were parameterized with three parameters for the hip and other three parameters for the knee joint in order to scale the amplitude, stretch the time and influence the period of the leg motion and change the amount of the hip/knee flexion and extension. Most vision systems use estimation algorithms while not capturing any actual data for angular position or ground contact. In force plate systems the subject has to calculate the step length in order to coincide with the sensing plate, otherwise no valid datas are generated. This could, of course, introduce bias in the measurements. Mechanically generated trajectories have a big limitation in on-line modifications, such as modifying step length and foot clearance.

According to Tucker et al. (2015) the human gait cycle can be segmented as a periodic sequence of phases or states, where specific events trigger transitions between phases. Quote: “*The choice of the number of states and the type of events used are somewhat arbitrary, and will depend on what information is available from the sensors and which joint the prosthesis or orthosis is to going actuate."* Iosa et al. (2013) propose a relationship between “*golden ratio"* and the proportion of consecutive gait phases, revealing an intrinsic harmonic structure of gait cycle. The Inertial Measurement Unit system implemented by Tunca et al. (2017) is capable of extracting a set of standard gait metrics like ground contact on and off, stride length, cadence, cycle time, stance time, swing time, stance ratio, speed, maximum/minimum clearance, and turning rate. With this information they can segment the gait cycle according to phases, phases prevalently found in the literature. Stöckel et al. (2015) propose eight functional periods (initial contact, loading response, mid stance, terminal stance, pre-swing, initial swing, mid swing, and terminal swing) distributed in two know phases (stance and swing). Most of these works have, on the one hand, common the ground contact triggered events (Heel Strike and Toe off) that are straightforward to detect. On the other hand the rest of the events are not that clearly distinguishable, and many rely on angle or time thresholds to find a measurable trigger event to switch between phases.

This paper, thus presents in section 2 both the overall mathematical method and the experimental setup used to validate the trajectory generator method. The section includes a brief description of the human-user test protocol and the group of subjects. In section 3 the work presents the experimental and statistical results. Section 4 follows with a discussion of the adequacy, saliency, and accuracy of the method as compared to the current state-of-the-art methods, and finally section 5 poses the concluding summary of this research and possible future directions.

## 2. Materials and Methods

In order to develop a method for systematically analyzing human gait data and for creating arbitrary gait trajectories for robotic rehabilitation systems, we begin by observing healthy subjects in controlled donning and use of an H2 robotic exo-skeleton while the robot is not powered. This section presents both the experimental protocol and experimental setup for user test with healthy subjects. The subject data can then be processed through mathematical techniques to obtain the geometric centroids and corresponding covariances of the location of characteristic key events found in the generalized gait cycle. The method then employs the centroids and corresponding confidence ellipses as polynomial spline nodes in the formulation, interpolation and scaling of a new ankle trajectory. The resulting trajectory is validated via conventional statistical methods. The ankle trajectory serves to formulate, via inverse kinematics, new joint angle control trajectories, thereby generating exo-skeleton limb gait of a desired step length.

### 2.1. Experimental Protocol

**Figure 1:**
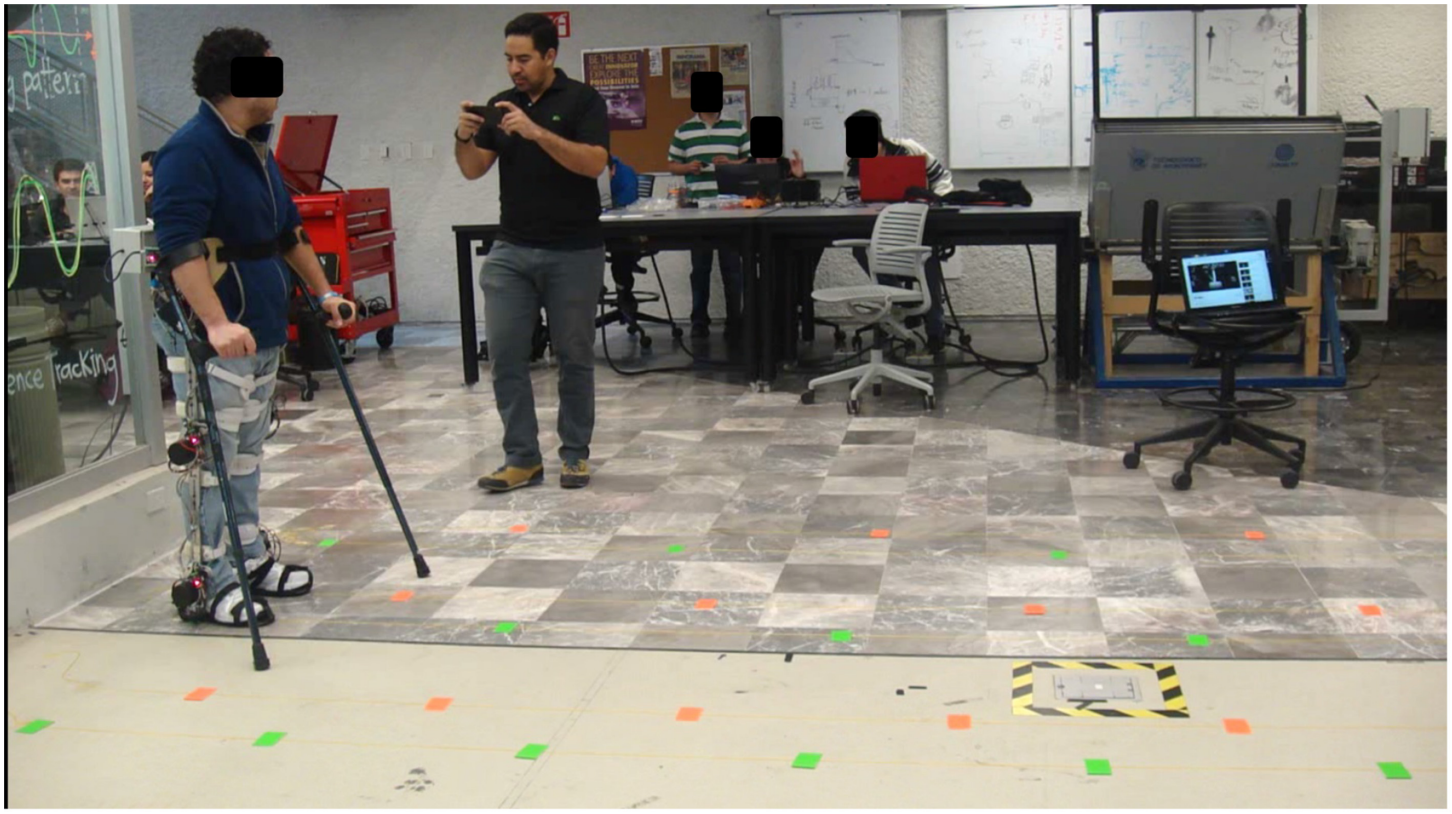
Experiment setup. The healthy subject is donning the exoskeleton and using crutches in order to replicate the case of use in rehabilitation. At the bottom there are colored marks on the floor making three lanes, one for each of three step lengths. Green marks are for the right foot and orange for the left one. The setup does not have to be modified during experimentation, as the marks in each lane are fixed.

Subjects are prompted to walk on flat terrain while donning the non-powered H2 lower limb exoskeleton, using crutches to replicate the conditions typically experienced by motor-impaired people when using an exoskeletal device. All tests are performed in a controlled laboratory environment. Each subject performs seven different trials with two repetitions each, at three step lengths and three step cadences, and one in free condition. The cadence and free conditions are not evaluated in the rest of the paper due to space. In all trials the healthy subject walks forwards, turns around, and returns on a 10 meter long track, thereby determining the number of steps during each particular trial. Two repetitions of each trial provides at least 60 gait cycles of each condition depending on the step length. Step length (*SL*) is defined by the distance from the point when the dominant heel makes contact with the ground to the next ground contact of the opposite heel. The step lengths (*SL*)s selected are 30, 45, and 60 [cm]. Each track was marked with sticky papers equidistantly for the length of the track as shown in Figure 1. Subjects are instructed to walk with the appropriate step length in order to step on each of the markers. The performed trials are described in Table 1 and the setup is shown in Figure 1.

**Table 1:** Test Description.

**Trial**	**Track**	**Trial description**	**Number of steps**
T1	Markers 30 cm	Self pace forwards and backwards 2 repetitions	16 steps
T2	Markers 45 cm	Self pace forwards and backwards 2 repetitions	12 steps
T3	Markers 60 cm	Self pace forwards and backwards 2 repetitions	8 steps

### 2.2. Experimental Setup

The H2 exoskeleton employed in this experiment was designed for rehabilitation protocols of adult patients with a height between 1.50 and 1.95[*m*], and with a maximum body weight of 100[*Kg*] (Bortole et al., 2015). The H2 is a robotic lower limb exoskeleton with six active powered joints, 3 per side, all operating in the sagittal plane, i.e., hip extension (−30°), hip flexion (+90°), knee flexion (+110°), knee extension (0°), ankle dorsiflexion (+20°), and ankle plantar flexion (−20°). Meaning that when the leg is fully extended, all 6 joints are in 0°of angular position. The exoskeleton has eight braces for attachment to the legs of the subject. One more brace is used to secure the exoskeleton structure to the subject's waist (see Figure 1). Each joint has its own electronics for low level control, i.e., motor spin with power electronics and network communication. Each joint has a brushless motor and a harmonic drive coupled to the leg segment of the exoskeleton in order to generate each movement. At the beginning of each segment a pair of strain gauges are located to measure the link deformation and therefore the interaction forces between device and user. Each joint has a pulley-base speed reducer and a precision potentiometer in order to capture the angular position of the joint. A pair of contact sensors, located under each foot sole, capture ground contacts. The first sensor is located under the heel and the second one is located under the distal pair of metatarsals.

Prior to the experiment, the motor transmissions are mechanically decoupled from all joints by removing the flexspline of the harmonic drive. The rest of the system remains unaltered to maintain the added mass of the exoskeleton but with reduced joint friction. In this way, the exoskeleton works in a fully passive mode, constraining the subject only from the kinematic and inertial points of view, and without applying any motive or resistive torques to subject joints i.e., motors are mechanically disconnected but the joint angle encoders are not. Additionally, each exoskeleton segment is adjusted and measured according to the length of the femur and tibia of each subject. The captured data thus contains the kinematics of the exoskeleton worn by the subject, including absolute angular position of hip, knee, and ankle joints from both sides. The strain gauge data is not captured since we assume that the friction force is negligible compared to the forces executed by the subject to move her legs and the exoskeleton. Furthermore, data from plantar contact sensors are also logged. Data are recorded at a frequency of 100 Hz and stored in an external SD Card as a log file.

### 2.3. Participant Subjects

Ten healthy subjects [5 males and 5 females, with 28 ± 6.56 (*SD*) year of age, 73 ± 9.04 (*SD*) kg, 1.71 ± 0.09 m, 8 right-handed and 2 left-handed] volunteered for the experiments. Lower-limb dominance was determined based on self-reported handedness, i.e., 8 RH and 2 LH. None of the subjects had symptoms of neurological or orthopedic dysfunction. Informed consent was obtained from all subjects according to the protocol of the Ethics Committee of the Tecnologico de Monterrey Robotics Laboratory.

### 2.4. Mathematical Methods

**Figure 2:**
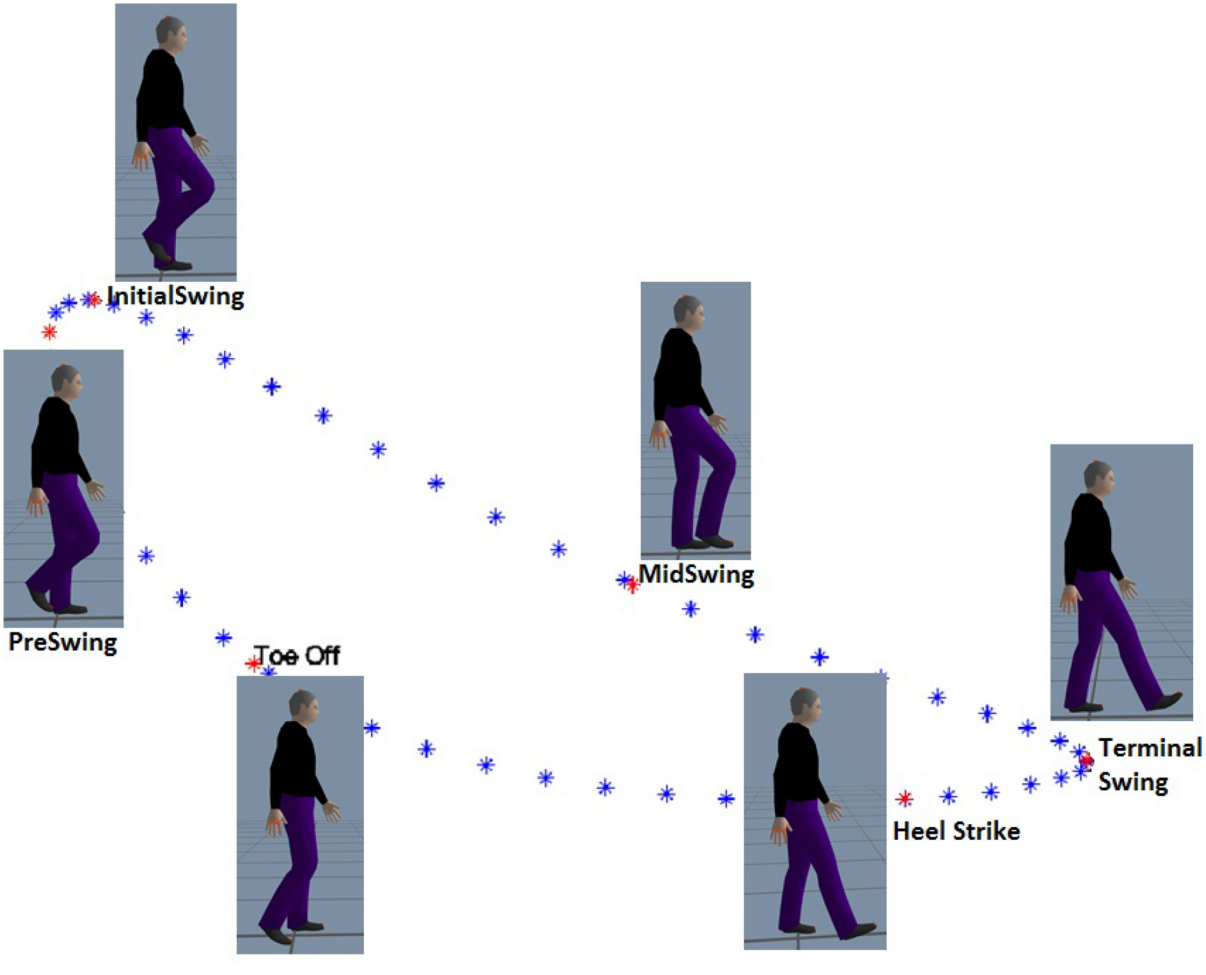
Defined key events are sequenced in order of occurrence: Heel Strike, Toe Off, Pre-Swing, Initial Swing, Mid-Swing, Terminal Swing. The position of the ankle in the sagittal plane is shown. Additionally, the approximate angular position of the hip and knee joints can also be seen in the cartoons.

The following subsections fully describe the mathematical method. The method captures gait data, identifies the subjects′ key events and generates control trajectories through the following steps: 1. Gait data capture (including ankle joint position localization in the sagittal plane based on direct kinematics and data identification and segmentation based on the well-known Heel Strike (HS) and Toe Off (TO) events - see Figure 2), 2. Centroid calculation to find a characteristic trajectory that represents the subjects′ walking pattern, 3. Trajectory generation based on modeled data, and 4. Comparison between captured and modeled data based on statistical analyses, whereby salient results were found among groups and subjects.

#### 2.4.1. Gait Data Capture

**Figure 3:**
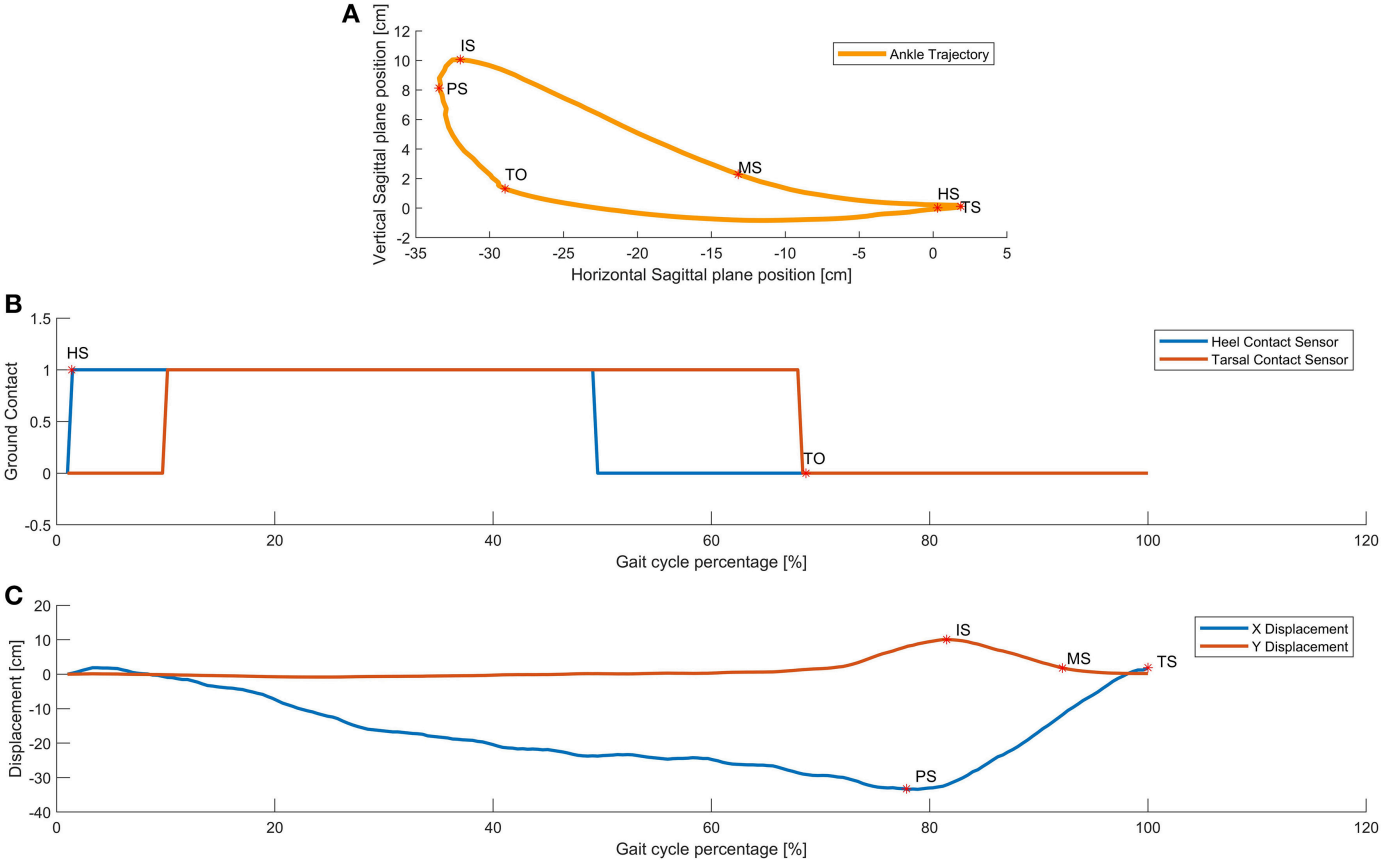
Walking events detection. In subfigure **(A)** Gait cycle key events calculated via forward kinematics of captured data. Subfigures **(B)** and **(C)**, show the ground contact and displacements of the ankle as functions of the percentace of the gait cycle. walking forward means moving left to right as gait evolves over time. The key events are determined as maximums and minimums identified by direction changes in velocity of the ankle.

Based on the recorded data as described in 2.2 and as the femur and tibia lengths are previously known for each subject, and since the angular positions for hip, knee and ankle joints are captured, these data are employed to reconstruct the leg movements by calculating the forward kinematics. For this calculation, hip-joint is the base and origin of the coordinate system while the ankle joint acts as the end effector for the kinematics model. From the series of forward kinematics from all gait cycles and repetitions of each trial per subject, ankle trajectories are recreated as shown in Figure 3A. In this way, ankle position in 2D space with respect to the hip was calculated in the sagittal plane. Although all the data for stride, i.e., both dominant and non-dominant sides is captured and stored, only the dominant limb data is employed in the analysis of the rest of this paper.

The captured data contains information about angular position of all three joints: hip, knee and ankle exoskeleton joints as well as contact sensors under heels and tarsals to detect ground contacts. The beginning of the step is defined by the Heel Strike *HS* event of the dominant limb defined as the moment that the heel makes contact with the ground. This event is detected by observing when the contact sensor magnitude changes from 0 *logic level* to 1 *logic level* or the rising edge as measured by two the instrumented insoles of the exoskeleton. These sensors are located in the most distal part of the metatarsals and just below the tarsal under both foot support plates of the exoskeleton. All of the data is segmented into steps using the following principle. Data of each step is normalized from 0 to 100% of the gait cycle, this means 0% (HS) is beginning of the step while 100% is the next HS of the same leg. The second event is called Toe Off *TO*, defined as the moment in which the toe is separating from the ground, detected by distal metatarsal contact sensor approximating 0 *logic level* or the falling edge and typically found at 60% of the step. Both events are shown in Figure 3B where pressure vs. step percentage was plotted.

While prior works (Iosa et al., 2013; Stöckel et al., 2015; Tucker et al., 2015; Tunca et al., 2017) have observed and analyzed the multiple phases of gait, we choose to identify events within the gait cycle, events that could have rigorous mathematical definitions. We propose that these points could serve as control nodes for splines that could then replicate the shape of gait patterns and trajectories. Due to the fact that the ankle trajectory has a kidney bean shape in the sagittal plane, as shown in Figure 3A, it was obvious that at least 4 control points would be necessary, if not more. With this in mind, a series of points was sought and observed. The gait literature already discusses two points considerably, namely heel strike and toe off as identified both in Figures 2 and 3.

A forward kinematics calculation provides the displacement of the ankle in both axes, i.e., vertical and horizontal vs. step percentage as shown in Figure 3C. With this information is possible to calculate both the first and second backward differences in order to also estimate the speed and acceleration, respectively. Using the first backward difference, it is possible to discover the zero velocity moments for both components of horizontal and vertical motion. The Equations (1, 2) represent both components, such that *x*_*i*_ is the actual observation, *x*_*i*−1_ is the previous observation, and Δ*t* is the time elapsed between samples.

(1) $$\begin{array}{l} ZVx_{i}=\frac{x_{i}-x_{i-1}}{\Delta t} \end{array}$$

(2) $$\begin{array}{l} ZVy_{i}=\frac{y_{i}-y_{i-1}}{\Delta t} \end{array}$$

In this context, zero velocity moments reflect a change of direction of the ankle joint displacement. The second backward difference shows acceleration and deceleration phases. Thus, two other relevant events are the maximum ankle displacement on the horizontal axis in both negative and positive directions, events herein termed Pre-Swing *PS* and Terminal Swing *TS*. Although some authors use these terms to describe phases of gait, we employ them to describe the instants when swing is at its upper and forward most locations.

**Figure 4:**
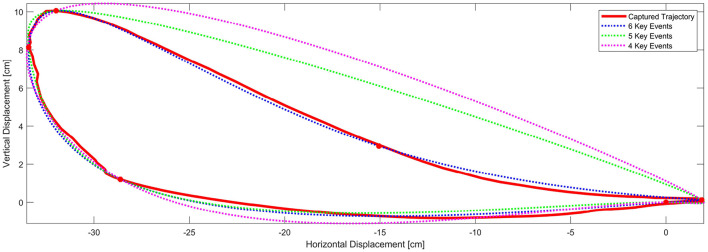
Comparison between using 4, 5 or 6 key events for the trajectory generator. Fidelity measures are: 72.4%, 81.7%, and 97.3%, respectively, thereby suggesting that the trajectory generator method should incorporate 6 key events herein proposed.

We wanted to know how closely the shape of the generated trajectories match the captured trajectory. By applying a statistical tool called Fidelity measure we were able to find out the grade of similarity for each combination. Figure 3C shows the position of the ankle joint over time in the horizontal axis where the change in slope is readily identifiable. In a similar way the first backward difference of displacement in the vertical axis allows the calculation of the Initial Swing *IS* instant. With these 5 key events, preliminary comparisons between the generated ankle trajectory and the captured trajectory, were visibly still not a close approximation. This can be summarized by the required six control points required to generate the kidney bean shape of the generated pattern. Figure 4 shows a comparison between 4, 5, and 6 key events trajectories and a random captured trajectory. We calculated Fidelity measures and obtained: 72.4%, 81.7%, and 97.3%, respectively, showing that there are significant differences in choosing <6 key events.

Therefore, we identify and acquire a sixth event, namely Mid Swing *MS*, defined as the location of the mean distance between the IS and TS events. These six events were calculated as previously described and placed in an arrangement for better data operations. It is worth emphasizing that the calculations of this stage were performed offline, i.e., after collecting all data from all subjects. From here onward the events will be referred to as the six key events and which are, in temporal order: HS, TO, PS, IS, MS, and TS as shown in Figures 2 and 3A.

(3) $$\begin{array}{l} P_{i}\left(x_{ik},y_{ik}\right),i=1,\ldots ,n,k=1,\ldots ,6 \end{array}$$

Where *P* is an array of points representing the key events, *x*_*j*_; *j* = 1, …, 6 represent the coordinates of the *jth* event, and *i* is the number of observations. Although the events are stored by their coordinates in the sagittal plane, they inherently contain their own time-stamp as a percentage of step. A sequence of ankle positions over time produces a closed ankle trajectory known as a captured trajectory. Thus the six key events of the gait sequence were identified (see Figure 3A). The method considers the hip joint as the origin of the coordinate system. All these six key events are detected in each step and appended into a labeled array for each subject. The key events found are represented in the sagittal plane coordinate system with the HS as the origin of the 2D plane to make the analysis more understandable and useful. The centroids are the average point of all key events of the same type and the same trial for all subjects. The representation of each key event per test was found as:

(4) $$\begin{array}{l} C_{j}=\left(\frac{1}{m}\sum x_{jk},\frac{1}{m}\sum y_{jk}\right),j=1,\ldots ,m,k=1,\ldots ,6 \end{array}$$

The *C*_*jk*_ pair represent the coordinates of the confidence hyper-ellipse centroid per trial i.e., there is one set of 6 centroids per trial, now using the HS event as origin of coordinates, with magnitude μ_*x*_ and μ_*y*_ with respect to the origin coordinates in the sagittal plane. Where *x*_*jk*_ and *y*_*jk*_ are the *j-th* centroid, *k* = 1 is HS, and continue with *TO, PS, IS, MS, TS*, μ_*x*_*jk*__ and μ_*y*_*jk*__ are the mean coordinates of the key events coordinates in their respective components. This means there exist clouds of points that represent the centroid of the key events found for each subject. *C*_*j*_ Represents the six centroids for each gait cycle, *j* is the *j-th* trial in the same order as described above. The gait cycle count depends on how many steps are made in the trial (not always the same due to segmentation, sometimes first or last steps are not taken into account, for 30[*cm*] trials between 15 and 17 steps, for 45[*cm*] trials between 10 and 13 steps and for 60[*cm*] trials between 8 and 19 steps are captured). This produces a new set of points for the key event centroids per each trial and test subject.

#### 2.4.2. Key Event Centroids and Dimensional Reduction

The EVD (Eigenvalue Decomposition) procedure is used as an orthogonal transformation to convert a set of observations of possibly correlated variables into a set of values of linearly uncorrelated variables. These variables correspond to the maximum variance in a specific axis. The number of eigenvalues is less than or equal to the smaller of the number of observations. This transformation is defined in such a way that the first component has the largest possible variance, and each succeeding component in turn has the highest variance possible under the constraint that it is orthogonal to the preceding components. The resulting vectors are an uncorrelated orthogonal basis set. Due to the nature of the data, the EVD process will result in two orthogonal axes rotated with respect to the original horizontal and vertical axes. In this way, it is possible to find the change in direction between the centroids per person and per event.

(5) $$\begin{array}{l} cov\left(X\right)=\frac{X^{T}X}{n-1} \end{array}$$

Where *X* is the data array with dimension *n* x 2 and *n* is the number of samples.

#### 2.4.3. Trajectory Scaling Direction and Interpolation

Using these same centroids, the variance magnitudes for each key event point cloud provide a resultant vector sum that adequately describes the scaling orientation whereby the trajectory can be modified via interpolation for other step sizes. This scaling represents a new axis rotated from the base coordinates. This way a vector and direction are found in order to scale the calculated trajectory as a function of the Step Length (SL). In order to calculate the scaling proportion, the reference step lengths from the trials can be employed. Taking, for example, the 30 cm SL trial as basis, and desiring a 35 cm SL, there exists a 17% increase, an increase to be scaled via the resultant direction of the linear regression between 30[*cm*] centroids and 60[*cm*] centroids for each key event. In addition to scale the foot clearance, the magnitude of the sum of the two eigen vectors for each centroid, represented the scaling direction, the same direction should be kept while increasing or decreasing the scale proportion.

The centroids of the 6 key events can now be employed as nodes to create a polynomial curve to represent the trajectories. For the closed interpolating curve, the first point of interpolation must be the same as the last point (Navidi, 2010). Each centroids will be employed as a nodes for the interpolation of the trajectory and represents both the starting point for the next polynomial curve and the ending point for the previous curve. Smoothness is ensured by requiring equality of both the first and second derivatives of the intersecting curves at each particular node as follows:

(6) $$\begin{array}{l} S_{0}=S_{n} \end{array}$$

In this way, the resulting trajectory is a closed and continuous ellipsoid curve. Each event that has been found produces a geometric point in the sagittal plane, called node. Each node is taken as a control point to generate the path for the calculated trajectory. Each node means a change in the function that defines the segment, each nodal transition is designed to be smooth meaning that the second derivative of one segment ending at the node is equal to the second derivative of the next segment starting at the same node. After obtaining the calculated trajectory as polynomials, the nodes can be parametrized based on the step length (*SL*). The calculated trajectory can then be modified based on the desired distance of *SL*.

By definition, a function *f*(*t*) is *C*^*k*^-continuous, if the function and its first *k* derivatives are continuous. Let *V* = {*t*_0_, …, *t*_*n*_}, *t*_*i*_ < *t*_*i* + 1_ the node vector by the event detector algorithm. To generate a periodic spline *S*, it is necessary to wrap the nodes *t*_0_ and *t*_*n*_ with each other, i.e., *S*(*t*_0_) = *S*(*t*_*n*_); $S^{\prime }\left(t_{0}\right)=S^{\prime }\left(t_{n}\right)$; $S^{\prime \prime }\left(t_{0}\right)=S^{\prime \prime }\left(t_{n}\right)$

The *S* spline has the coefficients *a*_*i*_, *b*_*i*_, *c*_*i*_, *d*_*i*_ in the shape of a cubic natural spline:

(7) $$\begin{array}{l} S\left(x\right)=\{\begin{array}{cc} S_{0}\left(x\right)=a_{0}x^{3}+b_{0}x^{2}+c_{0}x+d_{0}, & t_{0}⩽x⩽t_{1}\\ \vdots \\ S_{n-1}\left(x\right)=a_{n-1}x^{3}+b_{n-1}x^{2}+c_{n-1}x+d_{n-1}, & t_{n-1}⩽x⩽t_{n} \end{array} \end{array}$$

for *t* ∈ [*t*_*i*_, *t*_*i* + 1_], *i* = 0, …, *n* − 1

The calculated trajectory is a closed curve consisting of several segments. By definition, each segment has to be tangent the next, so each transition (key events denoted by the nodes) is “*smooth”* between segments.

#### 2.4.4. Statistical Validation of the Method

Multiple Analysis of variance (MANOVA) determines whether the means of three or more groups are different. The null hypothesis establishes that all of the population means are equal, whereas the alternative hypothesis establishes that at least one mean differs significantly from the rest. MANOVA uses *F*-tests to statistically test the equality of means. The F statistic compares the variability between the groups to the variability within the groups (Burden and Faires, 2010).

The groups are composed of the trajectory families for each one of the trials. They are grouped by participant and by trial. For example, all data for the 30 cm Step Length (SL) are grouped. Utilizing the MANOVA it is possible to find groups out within the same trial that are statistically different or not. For those groups of trials that the MANOVA fails to find similarities, this mean that the samples are rich in frequencies. This ensures that results are not biased. The independent variables are Step Length (SL), and Step Period (SP). Tukey′s method uncovers statistically significant differences between groups and permits a labeling of the most similar groups via simultaneous comparisons of the group means.

(8) $$\begin{array}{l} MST=\frac{\sum_{i=1}^{k}\left(T_{i}^{2}/n_{i}\right)-G^{2}/n}{k-1} \end{array}$$

(9) $$\begin{array}{l} MSE=\frac{\sum_{i=1}^{k}\sum_{j=1}^{n_{i}}Y_{ij}^{2}-\sum_{i=1}^{k}\left(T_{i}^{2}/n_{i}\right)}{k-1} \end{array}$$

(10) $$\begin{array}{l} F=\frac{MST}{MSE} \end{array}$$

Observations are single values of SL or SP. Groups are defined as sets of observations of the measures of SL and SP according to each test from the same subject. Where F is the variance ratio for Tukey′s test, MST is the mean square between groups, MSE is the mean square due to error (within groups), *n*_*i*_ is the number in group *i* and *n* is the total number of observations in the tested group, *T*_*i*_ is a group total, *Y*_*ij*_ is an observation, *G* is the grand total of all observations.

The Leave-One-Out Cross Validation (LOOCV) algorithm was used in order to estimate the level of fit of a single captured step (Test Trajectory) to the generated characteristic trajectory from the remaining dataset. This was done by leaving the test trajectory from the dataset out of the characteristic trajectory generation algorithm. Additionally the same process is repeated with the next single trajectory until completing the entire data set one by on. The core of the LOOCV algorithm is multiple comparisons that estimate how accurately a new characteristic trajectory will fit a new captured step.

(11) $$\begin{array}{l} N_{LOOCV30}=10\left(subjects\right)*4\left(trials\right)*15\left(steps\right)*14\\ \left(steps-1\right)=8400 \end{array}$$

(12) $$\begin{array}{l} N_{LOOCV45}=10\left(subjects\right)*4\left(trials\right)*9\left(steps\right)*8\\ \left(steps-1\right)=2880 \end{array}$$

(13) $$\begin{array}{l} N_{LOOCV60}=10\left(subjects\right)*4\left(trials\right)*7\left(steps\right)*6\\ \left(steps-1\right)=1680 \end{array}$$

This process must compare 8,400, 2,880 and 1,680 times for 30, 40, and 60[*cm*] trials, respectively, and compute the average for each trial set separately. The *Fidelity* measure servers to compare the trajectories to each other as presented by Cherelle et al. (2010).

(14) $$\begin{array}{l} Fidelity=\left(1-\frac{var\left(TestTrajectory-CharacteristicTrajectory\right)}{var\left(TestTrajectory\right)}\right)*100\% \end{array}$$

Since the number of samples of test trajectory may vary from one step to other, the characteristic trajectory has to be evaluated in the same number of samples in order to compute the point-to-point difference (*TestTrajectory* − *CharacteristicTrajectory*), then the variance of the resulting array has to be calculated and divided by the variance of the test Trajectory. The result is an estimation of how similar both trajectories are, the average contains the *N*_*LOOCVxx*_ Fidelity measures.

#### 2.4.5. Trajectory Step Length Scaling Process

Starting from all centroids found out for each trial, and taking into account the three centroids that belong to the same key event but in different trial, those represent points over the sagittal plane and it is possible to discover a line that minimizes de euclidean distance to the points applying linear regression. The resulting lines show that the same key events from the corresponding three characteristic trajectories are almost co-linear, e.g., *IS*_30_, *IS*_45_, *IS*_60_ almost coincide on the same line, proving that the corresponding Key Events to all steps length in between 30 and 60 [cm] move over these lines. The *HS* Key event is the origin of the coordinates so all of its centroids are aligned. With this information is possible to generate any trajectory in proportion to the actual variation in size starting from known sizes, the defined trials SL.

(15) $$\begin{array}{l} \begin{array}{c} desiredX_{i}=\frac{desiredStepSL-30}{30-45}*\left(C_{30}\left(i\right)-C_{45}\left(i\right)\right)+C_{30}\left(i\right)\\ desiredY_{i}=m_{i}*desiredX_{i}+b_{i} \end{array} \end{array}$$

Where *desiredStepSL* is the value in [cm] of the desired SL, 30 and 45 are trial SL, *C*_30_(*i*) and *C*_45_(*i*) are the centroid coordinates for 30 and 45 are trial SL, *m*_*i*_ is the slope and *b*_*i*_ is the Y-intercept of the corresponding line found by linear regression and *i* is the ith iterator for the key events in chronological order. To find the desired centroid coordinates for the new SL, the Equation 15 has been used. After applying this equation to the 6 key events, we knew six centroids that represent the starting points to run the generator algorithm and obtain a new trajectory with the desired SL.

## 3. Results

All six key events are identified in the captured trajectory, i.e., each gait cycle. Each key event conforms a dot cloud and its centroid is computed *HS* = (0.0, 0.0), *TO* = (−33.45, 2.92), *PS* = (−36.73, 6.12), *IS* = (−33.66, 9.45), *MS* = (−15.52, 3.52), *TS* = (2.61, 0.27). The centroids represent the key events found for all-subjects-mean gait cycle captured trajectories. A new set of events is calculated per trial, i.e., event identification and centroid computation for 30, 45, and 60 cm SL trials.

**Figure 5:**
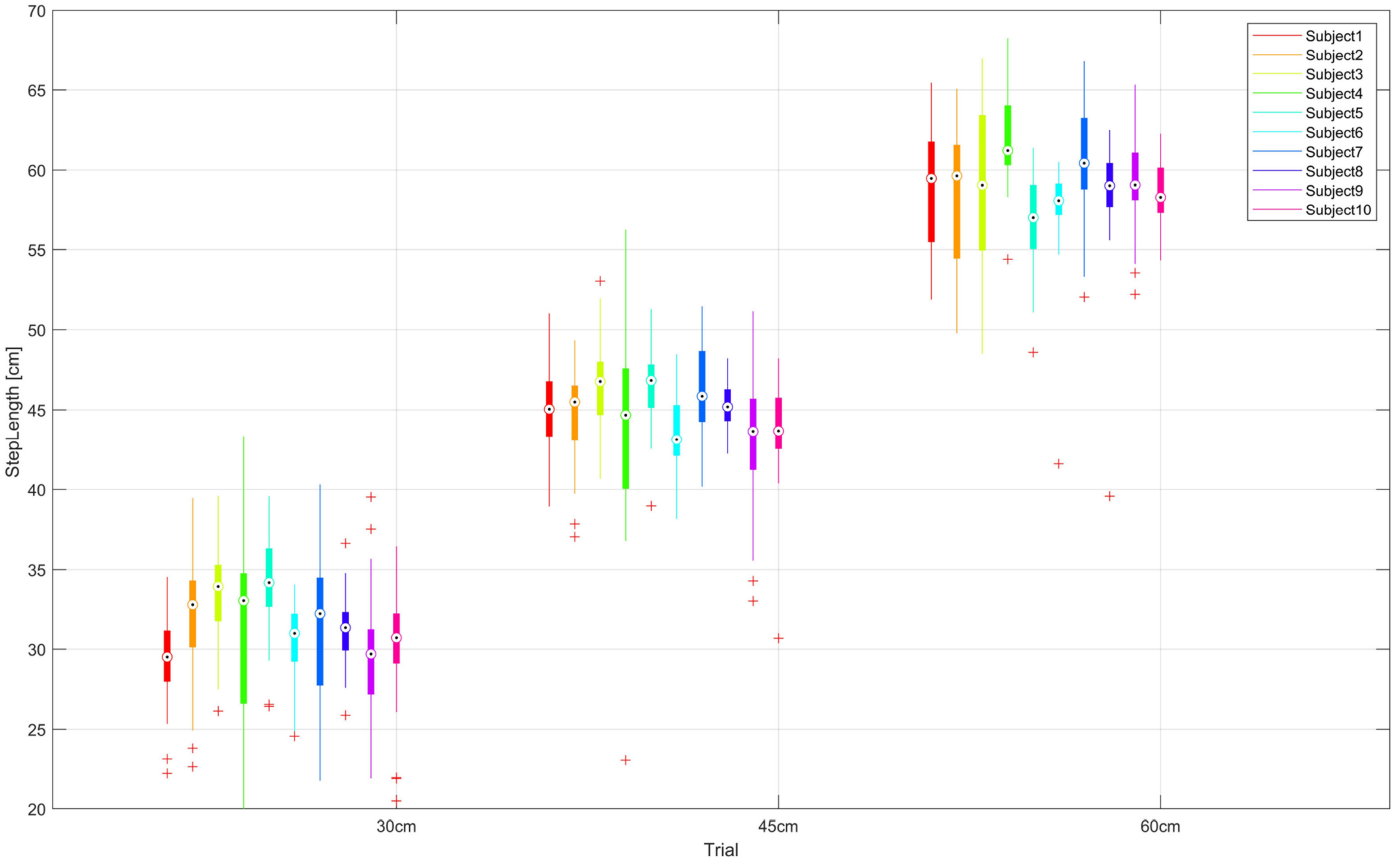
SL values are shown for each subject grouped per test. The median (dot), first and third quartiles (box), minimum and maximum values (lines) and some outliers (plus sign) are displayed. As expected, restricted SL trials appear close to each other across subjects, trials 1, 2, and 3.

The following Table 2 summarizes the actual data for Step Length (SL). The means and standard deviations for SL are calculated for all subjects per each trial. Expected reference values for each SL are covered by the mean and one σ standard deviation values in all cases, i.e., the 30[*cm*] reference value for SL is inside one σ of ${\bar{SL}}_{30cm}=31.262\pm 3.965\left[cm\right]$, for the 45[*cm*] reference value for SL is ${\bar{SL}}_{45cm}=44.873\pm 3.485\left[cm\right]$ and similarly for the 60[*cm*] reference value for SL is ${\bar{SL}}_{60cm}=58.882\pm 3.865\left[cm\right]$. These values show that subjects reasonably accomplished the tasks they were asked to do. In the three-SL trials (see Figure 5), the subjects perform steps of 30, 45, and 60 cm, as required. We observed certain variation across subjects (see Table 3), which confirms that performance of most subjects is different from each other.

**Table 2:** Actual data summarized for Step Length (SL) [cm] for all trials including all subjects.

	**30 cm**	**45 cm**	**60 cm**
SL	31.26± 3.96	44.87± 3.49	58.88± 3.87

**Table 3:** Grouping using the tukey method and 95% confidence for step length (SL).

**Trial 1 (SL = 30[cm])**							**Trial 2 (SL = 45[cm])**						**Trial 3 (SL = 60[cm])**					
Subject	N	Mean	Grouping				Subject	N	Mean	Grouping			Subject	N	Mean	Grouping		
S5	56	34.062	A				S3	27	46.714	A			S4	26	61.961	A		
S3	55	33.509	A				S5	34	46.415	A			S7	24	60.491	A	B	
S2	57	32.169	A	B			S7	34	46.377	A			S9	27	59.285	A	B	C
S8	56	31.259		B	C		S8	35	45.292	A	B		S1	28	58.882	A	B	C
S7	56	31.082		B	C		S1	36	45.085	A	B		S3	22	58.790	A	B	C
S6	56	30.670		B	C		S2	36	44.824	A	B		S10	27	58.657		B	C
S10	56	30.462		B	C		S4	36	44.260	A	B		S8	25	58.278		B	C
S4	55	30.198		B	C		S10	38	43.699		B		S2	26	58.212		B	C
S1	44	29.437			C		S6	33	43.505		B		S6	25	57.388		B	C
S9	57	29.417			C		S9	37	43.202		B		S5	26	56.893			C

**Figure 6:**
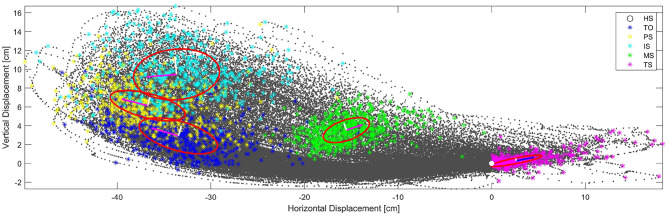
Gait cycle of all subjects aligned by HS for all repetitions of the trial *SL* = 30[*cm*]. Eigenvalue Decomposition for the centroids of Key Events. Heel Strike is placed in coordinates (0,0) and marked with a white cross. Toe Off events are shown in blue, *PS* are shown in yellow, *IS* are shown in cyan, *MS* are shown in green and *TS* are shown in magenta. Confidence ellipses are shown in red. Centroids and maximun and minimum variance are shown as vector components. Both *SL* = 45[*cm*] and *SL* = 60[*cm*] had similar results.

The components of the direction for the calculated trajectory can be represented by the EVD and confidence ellipse for each event. These components become the directions and proportions of scaling for modifying the calculated trajectories. (see Figure 6). Both orthogonal axes resulting from the EVD are the axes of maximum variance of the point cloud per event. In addition, major axis in red and minor axes in white indicate the proportion for the centroid to be moved in order to scale the calculated trajectory.

**Figure 7:**
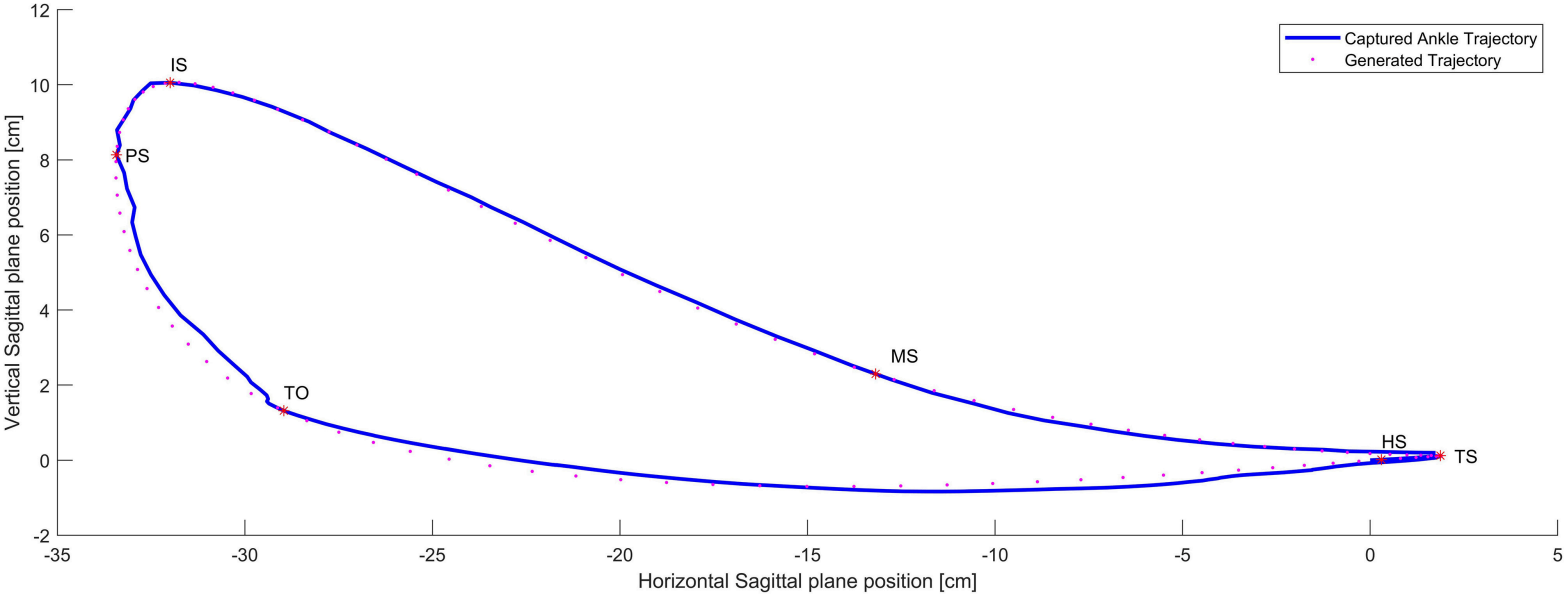
Captured (Full line) Vs Generated Trajectory (Dotted line). Fidelity 97.3% which is the degree of similarity of both trajectories.

Using cubic periodic splines based on the key events found in the calculated trajectory of the ankle joint of a healthy subject as shown in Figure 7 a polynomial segmented calculated trajectory was found. Thus, it is possible to generate the whole calculated trajectory using only the six key events as the control nodes.

A MANOVA test was run for SL data sets. Assuming the truth of the null hypothesis: all subject′s means are statistically equal in each trial. There is enough statistical evidence to reject the null hypothesis with a significance of α = 0.5 and *P* − *value* = 0.00. This means that at least one mean is statistically different from the rest of the trial group. Additionally, this proves that obtained data is statistically different between all users and enriches the data set for trajectory generation.

The Tukey method identifies any difference between two means that is greater than the expected standard error. Since the data is from 10 subjects, the Tukey test allows a paired test between all possible combination of subjects. The Tukey test generates a grouping map for the subjects with similar means for the SL trials (see Table 3). The simultaneous pairwise comparisons indicate that subjects without significant differences belong to the same group.

The results shown in Table 3 demonstrate that there are significant differences between some subjects, this contributes to form a data set that is rich in variations. Furthermore, this ensures that our experiments are not biased. As expected, every subject is different from every other, but at the same time some means are similar enough to be regrouped as smaller groups depending on the 95% confidence level. Finally, the data reveals that there is no question that the means of the all the trials across the different conditions are different in magnitude from each other. Thereby indicating that the methods can adequately differentiate step lengths.

**Figure 8:**
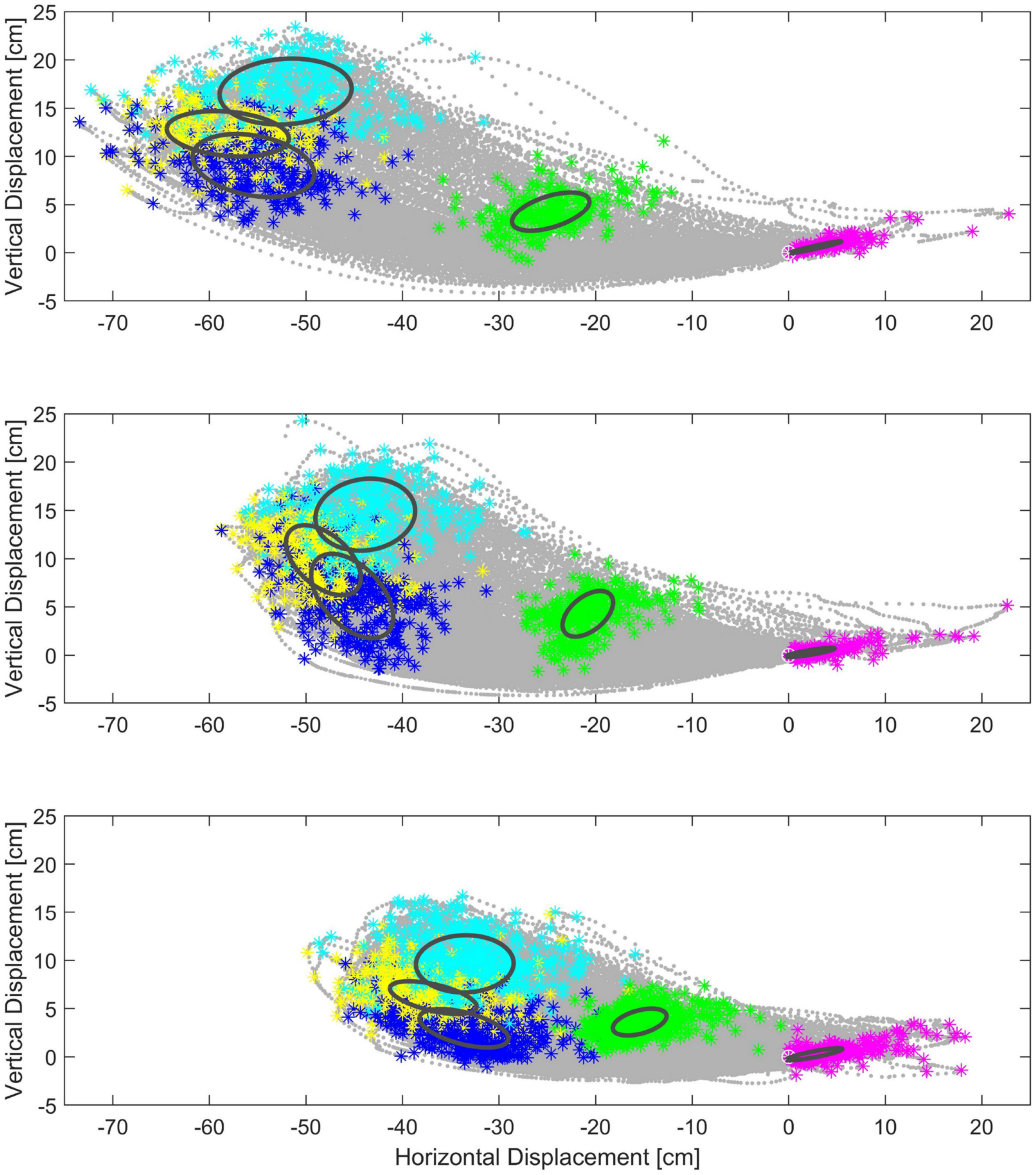
In the background, actual captured ankle position data point in the sagittal plane, all from subject gait cycles. Same Key events for all subjects are shown in color clusters. Centroids and ellipses represent average data and maximum and minimum variance per test. Top: Key events of 60[*cm*], Middle: Key events of 45[*cm*], Bottom: Key events of 30[*cm*].

Centroids of clustered data per each trial 30, 45, and 60 [cm] SL tests are shown, respectively, in (see Figure 8). With this we demonstrate that there actually significant differences between test. Confidence ellipses represents the region with 95% certainty according to a chi squared distribution to find a point that belongs to the corresponding key event region. On the one hand, it demonstrates that there exist significant differences between subjects, while on the other hand it is possible to group the points into elliptical regions.

**Figure 9:**
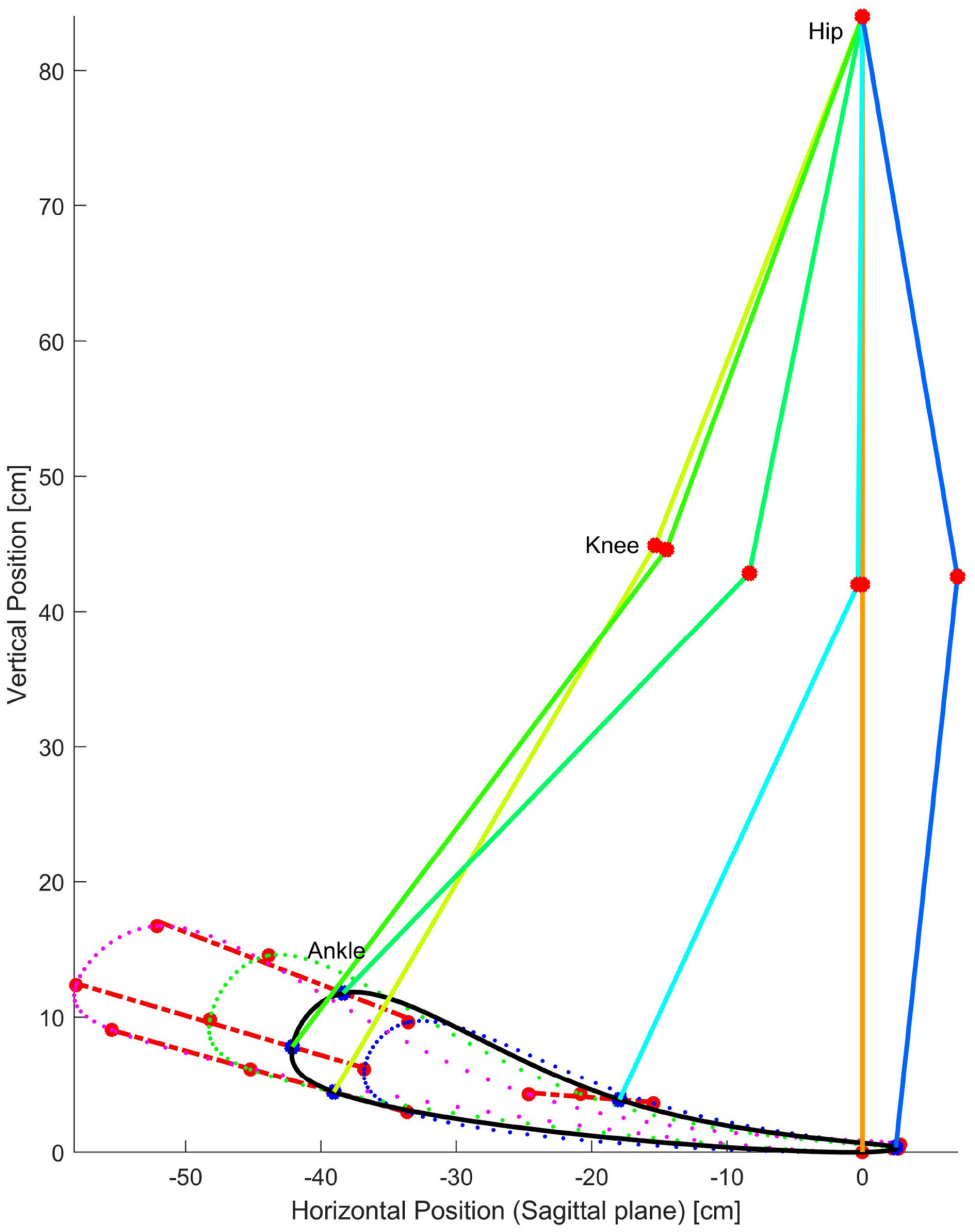
Generated characteristic trajectories in dotted lines for the three trials. Solid line represents the interpolated trajectory proposed for *SL* = 37[*cm*]. Product of the linear regression between centroids of the same key event, the red lines are used to represent the linear relationship between centroids for the same key event but different test. These lines suggest the use of linear interpolation to predict and set up any intermediate SL for programming a rehabilitation exoskleton as well as assistive devices. Angular position for hip and knee joints are calculated using inverse kinematics.

A comparison among the tests shows that centroids are almost aligned, linear fitting shows *R*^2^ value of $R_{TO}^{2}=0.9998$, $R_{PS}^{2}=0.9953$, $R_{IS}^{2}=0.9757$, $R_{MS}^{2}=0.8159$, $R_{TS}^{2}=0.4008$, (note that *HS* events are all aligned in the origin of coordinates). The mentioned linear fitting ${\bar{r}}_{i}$ represents a direction tendency for each key event. These tendencies can be used to interpolate an intermediate SL as needed. To interpolate a new trajectory, a point that belongs to ${\bar{r}}_{i}$ might be selected according to the desired scale (e.g., 37[*cm*] SL; see Figure 9).

Based on the six key events of the captured ankle trajectory as seen in the sagittal plane, a new calculated trajectory can be generated as needed. Average values of 95.2%, 96.1%, and 97.2% for 30, 45, and 60 [cm] SL trials including all subjects. These values reflect the trajectory fit and were revealed after running the LOOCV algorithm including the fidelity measure in each iteration.

The correct proportion and human likeness is the result of using the linear regression to propose new points over the line that represent the scale direction for each of the six key events. In Figure 9 a control trajectory was calculated for 37[*cm*], just in the middle of the first two trials to demonstrate that any reference trajectory could be generated between SL 30[*cm*] and SL 60[*cm*] the smallest and largest SL values for the present work.

## 4. Discussion

Prior work has attempted to apply healthy gait trajectories to exoskeletons being donned by disabled subjects, thereby forcing the subjects to follow a particular trajectory. This forced guidance causes the exoskeleton system to be uncomfortable. These healthy patterns neither deal with the limited range of motion nor take into account conditions typically addressed by disabled subjects when using an exoskeletal device. The introduction of the crutches and the non-powered exoskeleton in the data capture process provides adequate notions of the limitations of the joint ranges of motion, step lengths, foot clearances and step cadences. With this information, it could be possible to design rehabilitation gait trajectories with patient-specific variations between steps but still visibly human-like walking patterns.

Kazemi and Ozgoli (2018) introduced a real-time gait planner for human walking using a lower limb exoskeleton, in their work they present a feedback controller and pattern generator. Their work is based on two feedback-controlled third order systems as optimal trajectory planners for generating the walking trajectory, thereby adjusting the joint angles according to pre-configured parameters and stability margins. In contrast, this work requires both well-calculated parameters in order to converge in a limb trajectory and exact knowledge of the stablility margins.

Pre-programmed and constrained gait trajectories and controllers have been proposed previously. Gui et al. (2017) and Bhaumik et al. (2017) both presented predefined trajectories for lower limb exoskeletons, where trajectories tend to be fixed to a specific trajectory and followed by the controller named Central Pattern Generator and proprietary position controllers, respectively. In contrast to previous work that proposes pre-calculated lower limb trajectories, and little or no flexibility to adjust the trajectory according to the particular subject, we show that with knowledge of only six key-events of the gait cycle, it is possible to reconstruct a human-like ankle captured trajectory. This work introduces preliminary results for three reconstructed trajectories based on fixed SL trials. To generate a new calculated trajectory for an arbitrary desired SL, new key event locations could be selected from each direction tendency. The direction tendencies indicate the relative position of the new key events to the reference key events. The position of the new key events are over the direction tendency and proportional to the desired step in comparison with 30[*cm*] SL, i.e., change the SL to any length between 30 and 60[*cm*] to adapt the calculated trajectory to another subject.

In the present work a way to represent the ankle joint trajectories from only six key events has been studied. These key events have certain characteristics that makes them recognizable each time the “key events centroids and dimensional reduction" algorithm was run. The proposed representation for the trajectories allows to make a salient difference, comparison and evaluation of the ankle joint trajectories in the non-pathological walking cycle context. The first two key events are identifiable from ground contact sensors data. The remaining key events are unequivocally identifiable thanks to the position, velocity and acceleration during one walking cycle as described in 6. The definition of probability regions allows developers to make comparisons with different captured or new proposed trajectories. The benchmark evaluation of the captured or new proposed trajectories enables to test the relevance of a human-like walking cycle trajectory for the ankle.

For further comparison and experimentation a data base with captured raw and filtered data are available in: Mendoza et al. (2019). Data are organized per subject and trial for both raw and filtered logs. Please refer to the present publication for citations.

The present work does not include the testing of a control system for the joints in order to reproduce the generated trajectories with classical control techniques and thereby contrast the results to the reference trajectories. Furthermore, no method testing has been performed with impaired subjects. The method was also not tested in conditions of starting or ending a trajectory at any stage of the walking cycle. Besides no trajectories were based on a starting double foot support stance at any stage. And no trajectories considered conditions where the subject or therapist desired to begin, delay, or pause the therapy trial. All of these special conditions are left for future work.

## 5. Conclusion

This human-user experiment provided kinematic gait data, data collected during the walking of 10 healthy subjects in 7 different test conditions while they donned an instrumented, yet non-powered, exoskeleton. As a result of an initial data analysis looking at events of the gait cycle, this research proposes a novel mathematical method to analyze gait and generate limb-joint angle control trajectories primarily employed for therapeutic gait purposes in robotic rehabilitation systems. The method includes data acquisition and normalization, novel key event identification, and formulation of generated trajectories. The six key events that we propose and mathematically define, serve as the control nodes for a closed third-order polynomial spline to represent the ankle trajectory in the sagittal plane. The approach of considering the ankle joint as the end effector, gives the advantage being able to calculate a different reference trajectory on-line for each step each time as needed, i.e., adjust arbitrarily the step length, cadence, and/or foot clearance. This provides the controller the possibility of saliently adjusting the gait trajectories to different rehabilitation protocols by only modifying six parameters as key events. The main processor for the exoskeleton, an ARM micro-controller, could calculate the reference trajectory, the inverse kinematics, the angle references for each step, and generate angle references for all exoskeleton joints. The gait pattern parameters could also be set by the therapist according to experience and the patient′s therapeutic evolution.

The integrated method to capture, segment, and process data as well as to generate control trajectories demonstrated a throughput fitting error fidelity of 95.2, 96.1, and 97.2%, respectively, for each trial set. This is the result of the LOOCV test. This implies that the selected key events and the methods to manipulate them, minimize the quantity of required control reference data while increasing both trajectory saliency between patients and the ability to generate accurate new calculated trajectories for individual patients. Ultimately, it is possible to generate, with certainty, an arbitrary step length reference trajectory for any SL value between 30 and 60[*cm*]. Finally, the method proposed herein could accurately and saliently benchmark the human likeness of gait trajectories of existing robotic exoskeletal devices.

As stated by Leisman et al. (2016), “One may never have thought about how one plans and controls movement", and human locomotion is the result of a set of several mental processes. Motor actions are goal specific and integrate considerable cognitive functions to achieve successful motor performance. When one thinks about walking, one does not think about the particular angular position of lower limb joints. On contrary, one can consciously choose how long of a step to take. We took this approach on the trajectory generator for the ankle joint. Starting from the generated trajectory and then obtaining the corresponding joint angles.

## Ethics Statement

The human subject tests for the realization of this manuscript were approved by the Ethics Committee of the Tecnologico de Monterrey Robotics Laboratory. Since these tests were neither invasive nor introduced external power, human subjects agreed to participate. Written and informed consent was obtained from each participant.

## Author Contributions

RM-C and DT designed the experiments. RM-C collected, processed, and filtered data. Data interpretation was performed by RM-C, RS, and DT. RM-C drafted and wrote the manuscript. JH significantly improved the logic and structure of the manuscript. RS, JH, DT, JG, and JP reviewed the draft and made substantial comments. All authors have read and approved the final manuscript.

### Conflict of Interest Statement

The authors declare that the research was conducted in the absence of any commercial or financial relationships that could be construed as a potential conflict of interest.
